# The complete mitochondrial genome of *Graphomya rufitibia* (Diptera: Muscidae)

**DOI:** 10.1080/23802359.2018.1456375

**Published:** 2018-04-01

**Authors:** Wei Chen, Yanjie Shang, Lipin Ren, Xiangyan Zhang, Yadong Guo

**Affiliations:** Department of Forensic Science, School of Basic Medical Sciences, Central South University, Changsha, China

**Keywords:** Mitogenome, Muscidae, phylogenetic relationship, *Graphomya rufitibia*

## Abstract

*Graphomya rufitibia* (Diptera: Muscidae) was distributed worldwide. In this study, the complete mitogenome of *G. rufitibia* was sequenced and annotated, and the full-length was a 15,374 bp fragment, consisting of A (40.64%), G (8.96%), T (36.80%), and C (13.59%). The mitogenome is composed of 13 protein-coding genes, 22 transfer RNA genes, two ribosomal RNA genes, and a non-coding AT-rich region. Most PCGs used the canonical putative codon to start and stop, 21 tRNAs are folded into the classic clover-leaf structure, with the one exception of tRNA-Ser (AGN). A total length of 11,182 bp encoding 3727 amino acids, and account for 72.7% of the whole mitogenome. Phylogenetic analyses showed that *G. rufitibia* belongs to family Mydaeinae.

*Graphomya rufitibia* mainly spread over Asia and Australia, the larvae were semi-aquatic (Collin 1948; Arntfield 1975). It was reported that adult and larvae were coprophagous (Huang et al. 2007), thus bionomics of *G. rufitibia* making it was detrimental in animal husbandry and public health. Adults can be identified by the characteristic, but it is hard to distinguish in its young age, species identification by mitogenome is fast, economical, and reliable. More and more molecular studies about Muscidae have been published to supplement traditional taxonomy as rapid species identification ways.

Specimens of *G. rufitibia* were captured in August 2017 in Changsha city (28°12′N, 112°58′E), China. All voucher specimens were assigned a unique field code and reserved in the Guo’s Lab (Changsha city). The *G. rufitibia* mitogenome has been submitted to GenBank with accession number MG735216.

The complete mitogenome of *G. rufitibia* was amplified with 11 overlapping short PCR primers. PCR amplicons were conducted with the following procedures: initial denaturation at 94 °C for 4 min, followed by 35 cycles of 30 s at 94 °C, annealed at 50 °C for 30 s, and elongation 1 min at 72 °C, end in a final elongation for 10 min at 72 °C. PCR products were using an ABI PRISM 3730 automated sequencer (Applied Biosystems, Foster, CA) to sequence. The primitive sequence files were manually corrected for repeats at the beginning and end of the sequence to authorize a circular mitogenome, and then assembled into contigs with BioEdit 7.0.9.0 (Hall 1999). All encoding regions were manually aligned and validated. Open reading frames (ORFs) were executed through the NCBI ORF Finder according to the mitochondrial genetic codes of invertebrate. To make sure the authenticity of gene boundaries, all PCGs were compared with other muscid mitochondrial sequences under performed in MEGA 7.0 (Kumar et al. 2016).

Phylogenetic analyses of *G. rufitibia* with eight Muscidae species were performed based on 13 PCGs +2 rRNAs by using ML methods (Figure 1), and used two Sarcophagidae species as outgroups. Phylogenetic tree shows two clades, clade 1 consists of *Reinwardtiini* (*Muscina* genus) and Mydaeinae (*Graphomya* genus), which was corresponding to Kutty et al. (2014) research (Kutty et al. 2014). The clade 2 consists of Muscinae (*Stomoxyini*+* Muscini*), the topological structure was congruent with the previous studies (Kutty et al. 2014; Haseyama et al. 2015).

**Figure 1.**
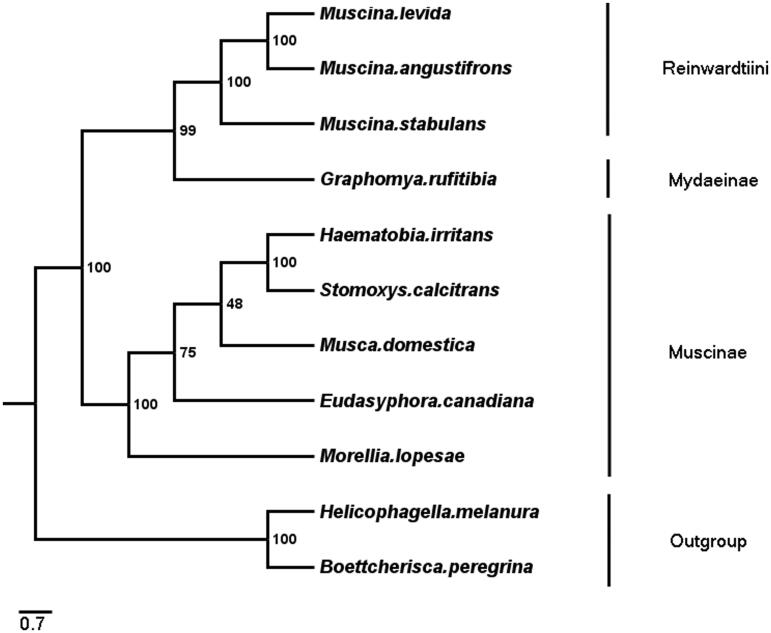
Phylogenetic trees of G. rufitibia with 8 species of Muscidae and two Sarcophagidae species as Outgroups, The tree based on 13 PCGs +2 rRNAs by using ML methods.
